# Supplementary material for: A Novel Squalenoylated Temozolomide Nanoparticle with Long Circulating Properties Reverses Drug Resistance in Glioblastoma
Source: Int J Mol Sci. 2025 May 15;26(10):4723. doi: 10.3390/ijms26104723 (PMC12112262; doi:10.3390/ijms26104723)
Supplement: Supplementary file 1 [file ijms-26-04723-s001.zip › ijms-3592002-supplementary.pdf]

Supplementary Information

The PDF file includes:

Figs. S1 to S9

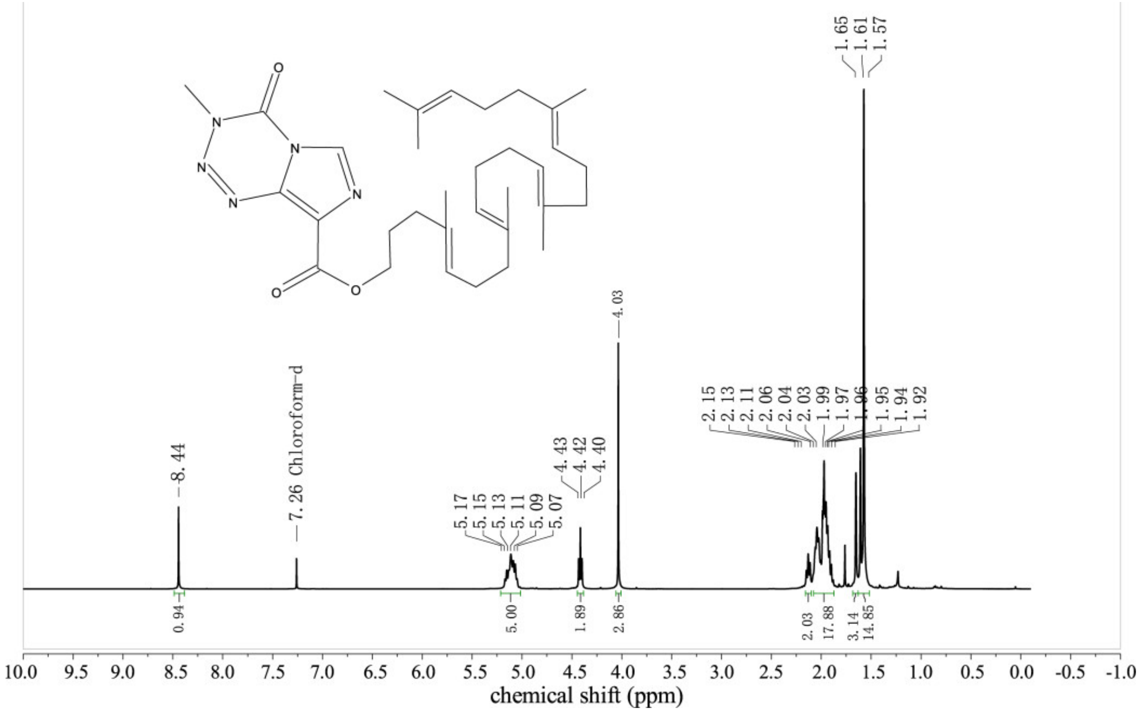

Figure S1. 1 H NMR spectra of SQ-TMZ.

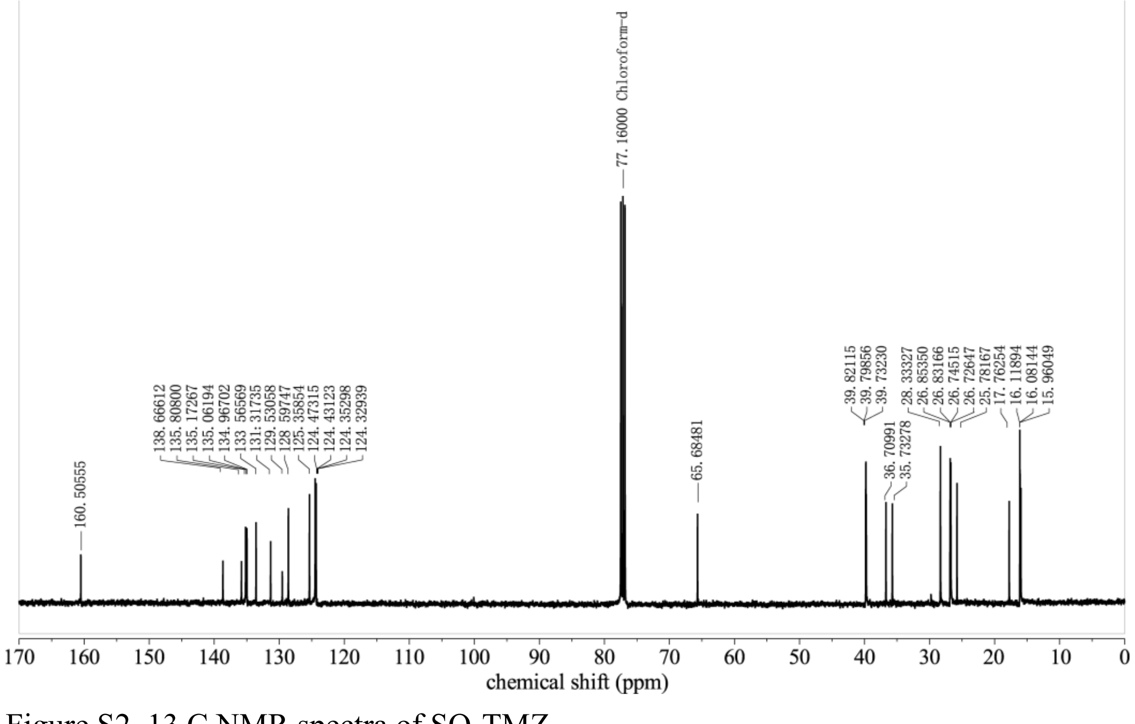

Figure S2. 13 C NMR spectra of SQ-TMZ.

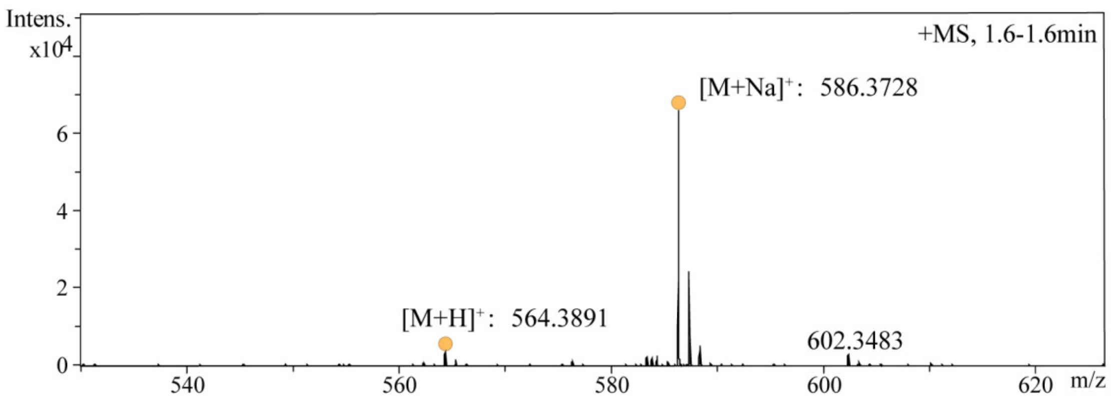

Figure S3. Mass spectrum. of SQ-TMZ.

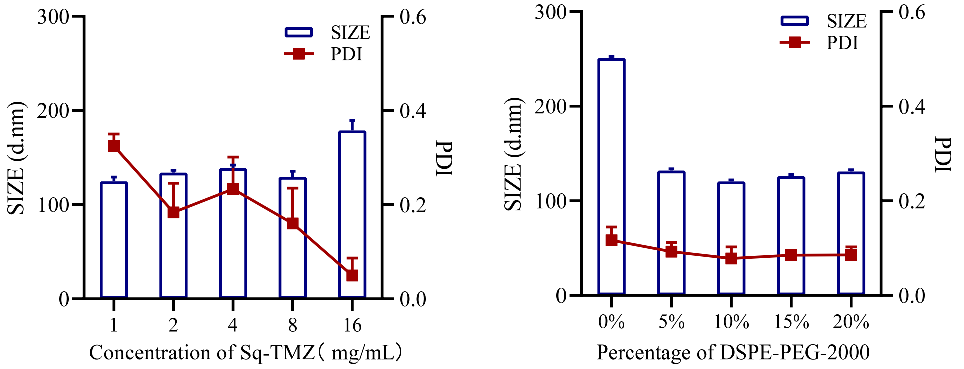

Figure S4. The effects of SQ-TMZ concentration (A) and DSPE-PEG-2000 content (B) on particle size and PDI. Mean ± SD, n=3.

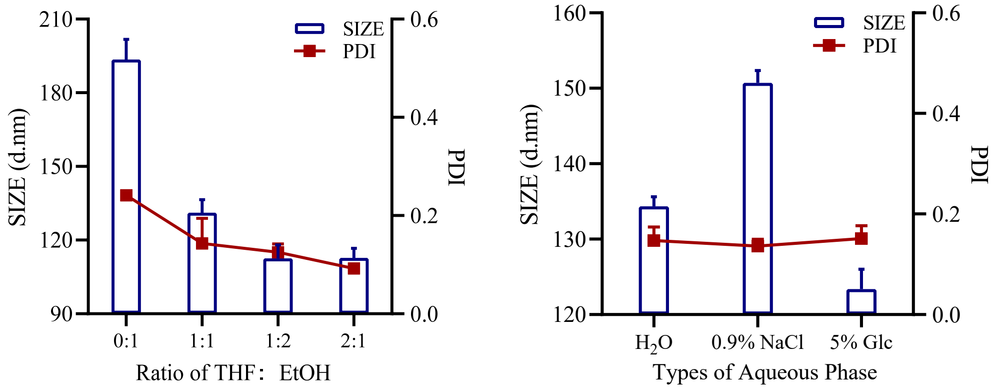

Figure S5. The effects of solvent ratio (A) and aqueous phase composition (B) on particle size and PDI. Mean ± SD, n=3

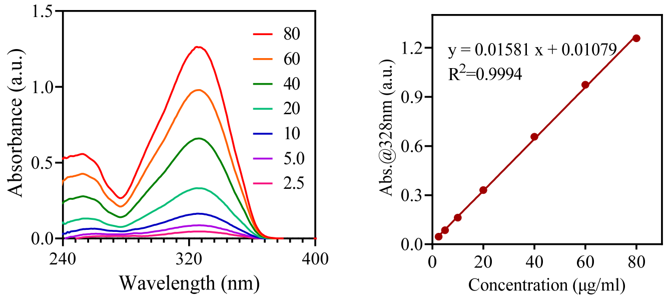

Figure S6. UV-Vis absorption (Abs) of different concentration of SQ-TMZ and the corresponding standard curve.

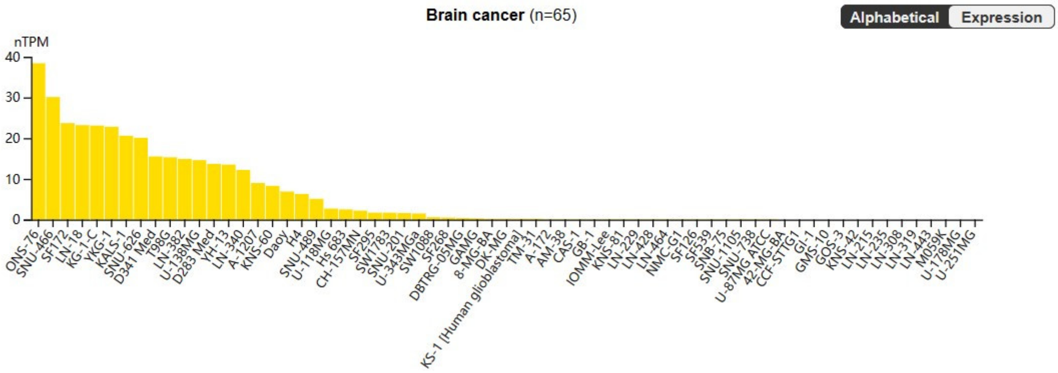

Figure S7. MGMT Expression in Brain Tumor Cell Lines from the HPA Database.

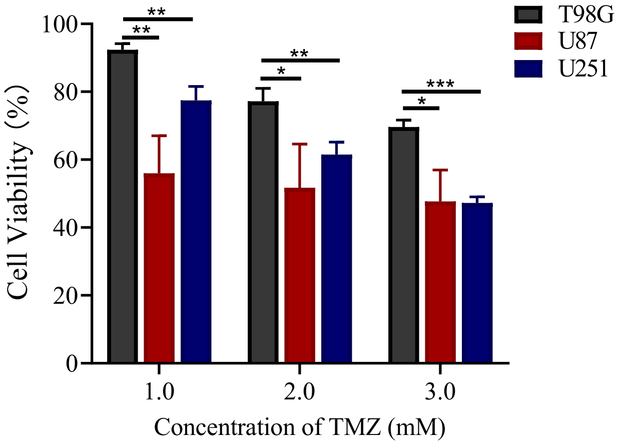

Figure S8. The sensitivity of T98G, U87 and U251 cells to TMZ. Mean ± SD, n = 3, t-test, ***P < 0.001, **P < 0.01, *P < 0.05.

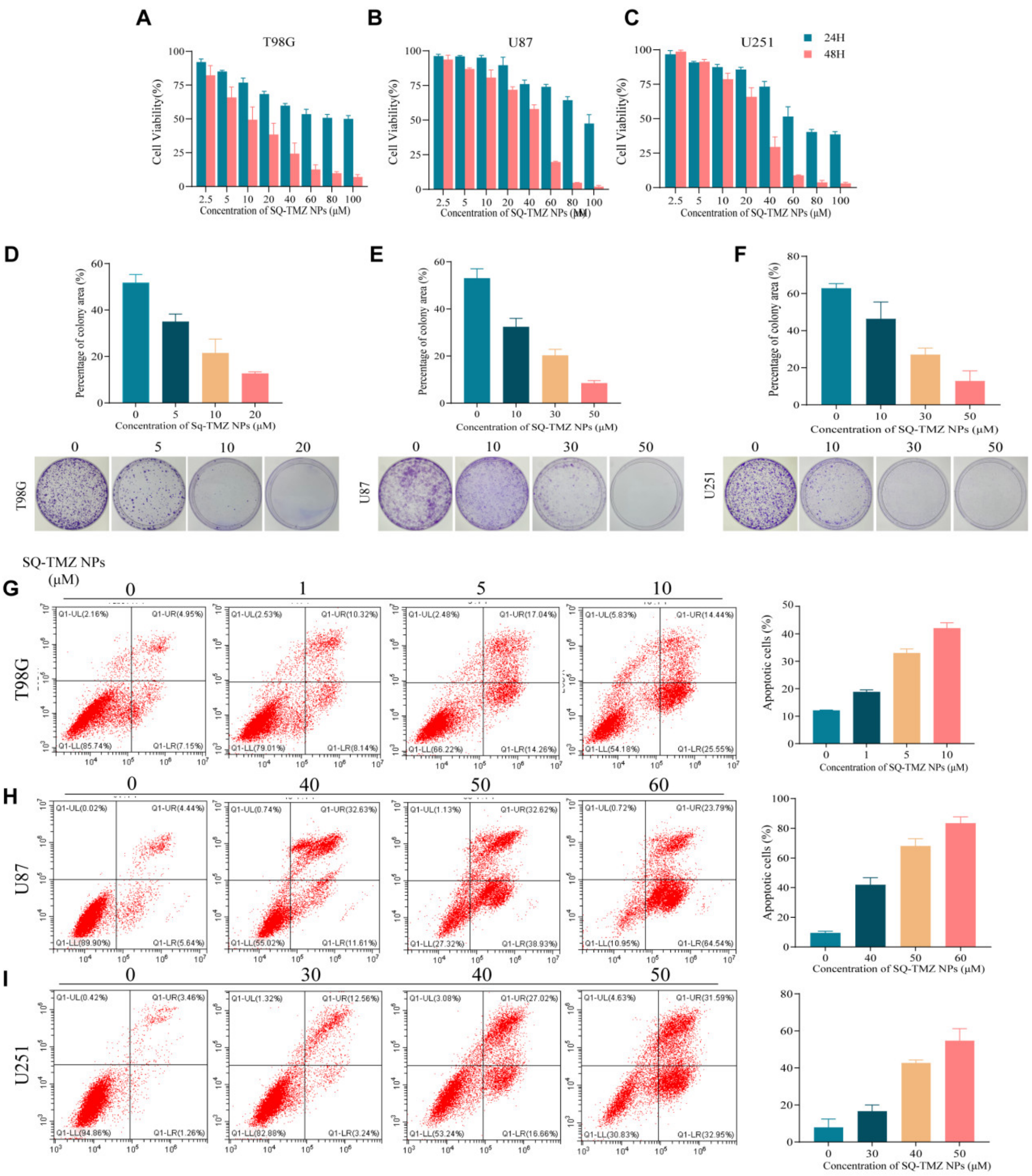

Figure S9. Toxicity study of SQ-TMZ NPs on T98G, U87, and U251 cells. (A, B, C) CCK-8 assay to evaluate the effect of SQ-TMZ NPs on the viability of the three cell lines. (D, E, F) Effect of SQ-TMZ NPs on the proliferation ability of the three cell lines. (G, H, I) Effect of SQ-TMZ NPs on the apoptosis of the three cell lines.
